# Terahertz spin-to-charge conversion in ferromagnetic Ni nanofilms

**DOI:** 10.1515/nanoph-2023-0089

**Published:** 2023-05-11

**Authors:** Hao Cheng, Yangkai Wang, Zheng Liu, Xiangyu Jia, Qiuping Huang, Yalin Lu

**Affiliations:** Department of Materials Science and Engineering, University of Science and Technology of China, Hefei 230026, P.R. China; Hefei National Research Center for Physical Sciences at the Microscale, Anhui Laboratory of Advanced Photon Science and Technology, University of Science and Technology of China, Hefei 230026, P.R. China

**Keywords:** ferromagnetic films, inverse spin Hall effect, spin-to-charge conversion, spintronic terahertz emitters

## Abstract

Spintronic terahertz (THz) emission via spin-to-charge conversion (SCC) has been widely studied in ferromagnets (FM)/nonmagnets (NM) structures, in which various mechanisms of SCC have been confirmed in different NM materials. However, it is rare to find a material having multiple SCC mechanisms at the same time. Here, we report a ferromagnetic metal Ni film with diverse functions in the SCC process, by performing THz emission experiments in single Ni layer, FM/Ni, Ni/NM bilayers and FM/Ni/NM trilayers. It is demonstrated that in Ni monolayer, THz emission is radiated by the anomalous Hall effect and ultrafast demagnetization of Ni film. In FM/Ni, the Ni film acts as an SCC implementer and THz emission is mainly generated by the inverse spin Hall effect (ISHE) of Ni. In Ni/NM, the Ni film acts as a spin injector and provides spin currents to be converted to charge current via ISHE of heavy metal NM, inducing THz emission. In FM/Ni/NM, THz emission mainly comes from ISHE of FM/Ni, Ni/NM, and FM/NM, and their domination is relative to Ni thickness. Our findings show a ferromagnetic film not only acts as a spin injector but also as an SCC implementer, providing a new concept to design spintronic THz emitters.

## 1 Introduction

Terahertz waves (0.1–10 THz) show frequencies in the range of resonance window of many materials and have potential applications in materials analysis, imaging, communication and spectroscopy [1–4]. The development of THz science and technology is strongly dependent on the high-efficiency sources, high-sensitivity detectors and functional components. Among them, seeking for a stable and efficient source is the most crucial one. The common THz radiation can be generated by optical rectification from electro-optical (EO) crystals [5–7] and transient electrical currents in semiconductor antennas [8–11] and so on. However, most semiconductors and EO crystals suffer from the optical phonon resonances, which strongly absorb the THz radiation and thus severely affect the bandwidth [12–14]. Recently, spintronic THz emission based on spin-to-charge conversion (SCC) has obtained a great deal of attention due to its advantages of low cost, broad bandwidth, easy fabrication and flexibility [15–25]. The basic structures of spintronic THz emitters are ferromagnetic (FM) and nonmagnetic (NM) metal thin film heterostructures [15], [16], [17, 20]. The key to spintronic THz emission is the SCC process. The femtosecond laser pulse excites the metal stack and induces non-equilibrium spin polarized electrons through ultrafast superdiffusive spin currents [26–28] or ultrafast spin Seebeck effect [29–31]. The spin currents perpendicular to the plane diffuse to the nonmagnetic metal layer, and then form an in-plane ultrafast charge current emitting THz electromagnetic wave. This SCC process is usually converted by the inverse spin Hall effect (ISHE) [32–34] or inverse Rashba–Edelstein effect (IREE) [22, 35, 36]. The former occurs in the bulk of nonmagnetic metals while the later occurs in the regions with broken inversion symmetry like interfaces [36].

In order to enhance the performance of spintronic THz emitter, various materials have been adopted via different SCC mechanisms [16, 20, 22, 28, 36], [37], [38], [39]. At the beginning, the nonmagnetic metal materials used in the spintronic emitters are heavy metals like Pt and W [16]. Those heavy metals radiate THz radiation efficiently via the ISHE because they have large spin Hall angle (SHA) due to their strong spin–orbit coupling. Later, different types of materials with SCC were studied, such as topological insulators [40], semiconductors [38, 41] and even light metals [25, 42]. To enhance the SCC efficiency, the researchers start to study the interface effect. For example, the interface of Ag and Bi exhibits the SCC via IREE in the THz regime [22, 36]. Recently, single-layer ferromagnet has been demonstrated to generate THz emission resulting from the interfacial effects via anomalous Hall effect (AHE) [43]. Aside from the AHE, ultrafast demagnetization also can arise the THz emission in single-layer ferromagnet [44–46]. More recently, efficient SCC of nonmagnetic metal materials with small SHA like Cu and Al is studied and its mechanism is demonstrated to be interfacial skew scattering [28]. So far, diverse mechanisms of THz emission via SCC have been confirmed in different materials respectively. Among the available results, one spintronic THz emitter is corresponding to the lone SCC mechanism. It is rare to have those THz emission mechanisms in one material at the same time. Therefore, it is highly interesting to further explore a material having multiple functions in the SCC process, which can provide a new concept to design the spintronic THz emitter.

Although the self-induced charge currents in ferromagnets like Ni_80_Fe_20_ (Py), Fe, Co and Ni by the inverse spin Hall effect have been probed in previous works [47–51]. Those charge currents in ferromagnets have not been studied in frequency of THz. Actually, unlike the NM metals, those FM metals can be used not only as spin injectors, but also as spin detectors. Therefore, in this paper, we study the THz emission via SCC of ferromagnetic metal Ni film in different structures: single Ni film, FM/Ni bilayer and Ni/NM bilayer, respectively. We find the Ni film plays different roles and the THz radiation mechanisms are also different in those structure. We also try to integrate those mechanisms in one structure and provide a theoretical way to enhance the SCC efficiency. This work indicates that many other ferromagnetic metals may have potential to enhance the SCC and may be exploited for application in spintronic THz emitters.

## 2 Results and discussion

### 2.1 Terahertz spin-to-charge conversion in Ni monolayer

**Figure 1:**
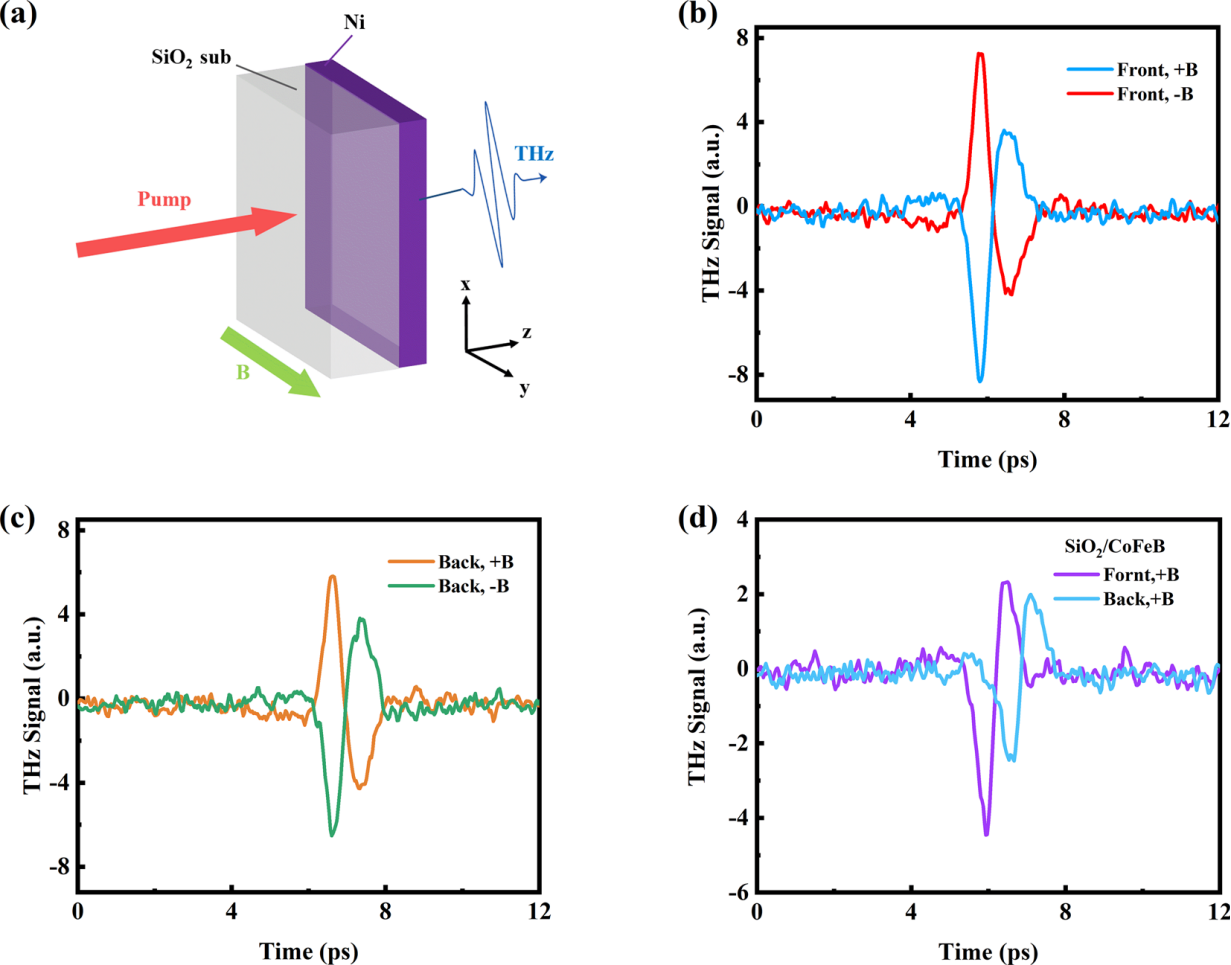
THz emission from single Ni layer. (a) Schematic of THz emission from SiO_2_/Ni structure. (b) Typical THz emission from SiO_2_/Ni (4 nm) structure with reversed magnetic fields when pumping from the SiO_2_ substrate side (front). (c) THz emission from SiO_2_/Ni structure with reversed magnetic fields when pumping from the Ni film side (back). (d) THz emission from SiO_2_/CoFeB (4 nm) structure with flipping the sample in positive magnetic field.

The schematic of THz wave generated from single Ni layer is shown in Figure 1(a). A 4 nm Ni layer was deposited on the SiO_2_ substrate by magnetron sputtering. A femtosecond laser pulse excites the Ni film and triggers a spin current. Figure 1(b) and (c) show the typical waveforms of THz electric field emitted from the Ni film. It is apparent that the polarity of the emitted THz waves reverses when the applied magnetic field is reversed, indicating that the THz pluses are dependent on the magnetic origin. The THz emission from single layer ferromagnetic metal can be owning to ultrafast demagnetization [45] and interfacial effect via anomalous Hall effect (AHE) [43]. And the THz emission caused by the ultrafast magnetization is described by the following equation [44]:

(1) $$E_{x}\left(t\right)=\frac{\mu_{0}}{4\pi^{2}r}\frac{\partial^{2}M_{y}}{\partial t^{2}}\left(t-r/c\right),$$

where ***E***_*x*_ is the *x*-component of THz electric field, *r* is the distance to the magnetic dipole, *μ*_0_ is the vacuum permeability, and *c* is the speed of light. ***M***_*y*_ is the *y*-component of magnetization. For ultrafast magnetization, the THz emission from the sample is determined by the second derivative of the magnetization with time, so the THz wave is independent on the stack order of the ferromagnetic metal and the substrate.

However, when changing the pump direction from the SiO_2_ substrate side to the Ni film side, the THz wave polarity is also reversed. This behavior is opposite to that of SiO_2_/CoFeB (4 nm) structure in Figure 1(d). In SiO_2_/CoFeB structure, the THz wave polarity is independent on the FM/dielectric interface stacking order, implying that the THz emission from SiO_2_/CoFeB is mainly attributed to the ultrafast magnetization. However, the THz emission from SiO_2_/Ni structure is relative to the FM/dielectric interface stacking order when keeping the direction of the external magnetic field unchanged. That means the THz emission from Ni/SiO_2_ substrate may not just result from the ultrafast demagnetization. By the way, the time delay of THz emissions is different when exciting the samples from the side of the substrate and the films, which originates from the different refractive indices of SiO_2_ substrate in 800 nm and THz frequency range.

Those behaviors are similar to the THz signal from the FM/NM bilayer. In the FM/NM emitter, the reversal of spin current direction is responsible for the reversal polarization of THz emission when flipping the sample. However, in single layer of Ni film, it does not exist because flipping the sample does not break the symmetry and change the spin current direction. The THz signal reversal may be caused by the presence of AHE. The longitudinal current ***j***_l_ is converted to a transient transverse current ***j***_t_ by the AHE [43]:

(2) $$j_{\text{t}}=\theta_{\text{AHE}}M\times j_{\text{l}},$$

where ***M*** is the magnetization direction, and *θ*_AHE_ is the AHE angle. Because ***j***_t_ is related to the Ni/SiO_2_ interface and magnetic direction, when flipping the sample or reversing magnetic direction, ***j***_t_ is reversed.

**Figure 2:**
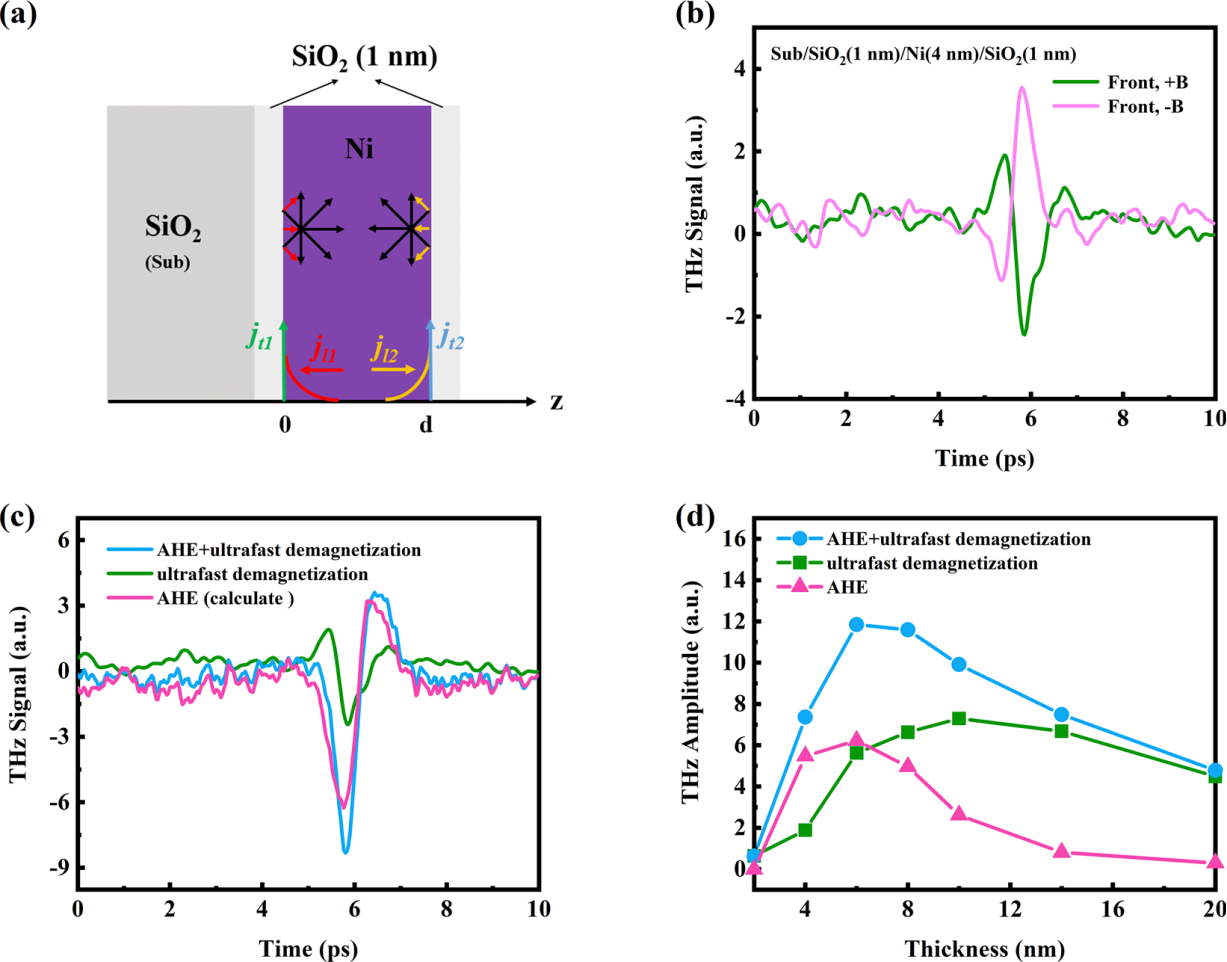
Separating the THz emission from AHE and ultrafast demagnetization in single Ni layer. (a) Schematic of THz emission from single Ni film caused by interfacial effect via AHE. (b) THz emission from Sub/SiO_2_(1 nm)/Ni(4 nm)/SiO_2_ (1 nm) sample. (c) THz wave from co-effect of AHE and ultrafast demagnetization (blue line), ultrafast demagnetization (green line) and calculated AHE signal. (d) THz intensity as a function of Ni thickness contributed by AHE (pink), ultrafast demagnetization (green) and their co-effect (blue).

Therefore, the interfacial effect via AHE dominates the THz emission from the single 4 nm Ni film. As shown in Figure 2(a), the process can be simply described as follows [43]: (1) the fs laser irradiates the Ni film and generates nonequilibrium electrons. (2) The nonequilibrium electrons diffuse in a random direction until they suffer a scattering and are bounced back in any random direction at the Ni/dielectric interface, forming backflow currents from the bottom (***j***_l1_) and top (***j***_l2_) interfaces, leading to a net backflow current (***j***_l1_ − ***j***_l2_) due to the discrepancy of two interface. (3) This backflow current is then converted into a transvers transient current due to the large AHE of Ni film, emitting THz radiation.

Therefore, the THz emission from the single Ni layer is the co-effect of AHE and ultrafast demagnetization. This explains why the THz amplitudes are different when the laser excites in different side of the sample. When the laser beam is incident from the substrate side, the THz wave polarities are the same for two mechanisms, resulting in an enhanced THz radiation; while the laser irradiates the sample from the metal side, the THz wave polarities are opposite, thus, reducing the THz signal.

However, if the bottom and top interfaces of Ni film are the same, the backflow currents ***j***_l1_ and ***j***_l2_ are cancelled out. So the contribute of AHE part is suppressed even vanished. At this point, we could separate the contribution of AHE and ultrafast demagnetization in single layer Ni. We deposited a 1 nm SiO_2_ film on the top of Ni layer, and introduced a new interface (top interface). To make the bottom and top interface as same as possible, we firstly deposited a 1 nm SiO_2_ film on the substrate, then deposited the Ni film and 1 nm top SiO_2_ film. However, the bottom and top interfaces cannot be completely the same, and the AHE radiation cannot be completely eliminated. But, in this way, we can eliminate most of the AHE contribution. So we consider the THz wave emitted from the Sub/SiO_2_(1 nm)/Ni/SiO_2_ (1 nm) sample to be almost caused by the ultrafast demagnetization. And the THz intensity from this sample is the ultrafast demagnetization contribution.

The THz wave emitted from Sub/SiO_2_(1 nm)/Ni(4 nm)/SiO_2_ (1 nm) sample is shown in Figure 2(b). We find that the THz signal is reduced because the AHE radiation is suppressed. As the Figure 1(b) shows the THz wave from the single Ni layer, it is induced by the co-effect of AHE and ultrafast demagnetization. The THz intensity discrepancy of two figures is the AHE contribution. In Figure 2(c), We use the THz wave from sample of Sub/Ni(4 nm) (blue line) to deduct the contribution of ultrafast demagnetization (green line) and plot the THz signal of the AHE contribution (pink line). In this way, we deposited a series of Sub/SiO_2_(1 nm)/Ni(*d*_Ni_)/SiO_2_ (1 nm) samples, and drew the Ni thickness dependence of THz intensity from contributions of AHE and ultrafast demagnetization, respectively. As is shown in Figure 2(d), for AHE contribution, the THz intensity reaches its maximum value at about 6 nm of Ni and then decreases gradually. This result is consistent with the work in Ref [43]. For the ultrafast demagnetization contribution, the THz emission increases with the Ni thickness within 10 nm, then decreases slowly when the Ni thickness beyond 10 nm. This can be explained by the reason that magnetism increases with the Ni thickness increases. But when the Ni thickness increases, the absorption of THz wave by the metal film also increases. In general, when the Ni thickness is between 2 and 6 nm, the contribution of AHE is dominant. When the Ni thickness over 8 nm, the contribution of ultrafast demagnetization is dominant.

In short, the THz emission could be emitted from the single Ni thin film, and its mechanism is the co-effect of the AHE and ultrafast demagnetization. Moreover, when the Ni thickness is less than 6 nm, AHE dominates. On the contrary, the ultrafast demagnetization is dominant. But the strength of the THz radiation is quite small compared to the common FM/NM spintronic THz emitters. Thus, we further study more THz emission from bilayer structures composed of Ni film to enhance the SCC.

### 2.2 Terahertz emission from FM/Ni structure

As we know, the Ni is a ferromagnet, and it is rarely reported about the THz radiation emitted from FM/FM structures. In our previous work, we demonstrated efficient THz emission from CoFeB/NiCu heterostructure with phase-transition metal NiCu either in its paramagnetic state or ferromagnetic state [25]. Therefore, it is very possible to generate THz emission from the FM/Ni structure at room temperature. In this part, we studied THz wave emitted by SCC in the structure of CoFeB/Ni bilayer.

**Figure 3:**
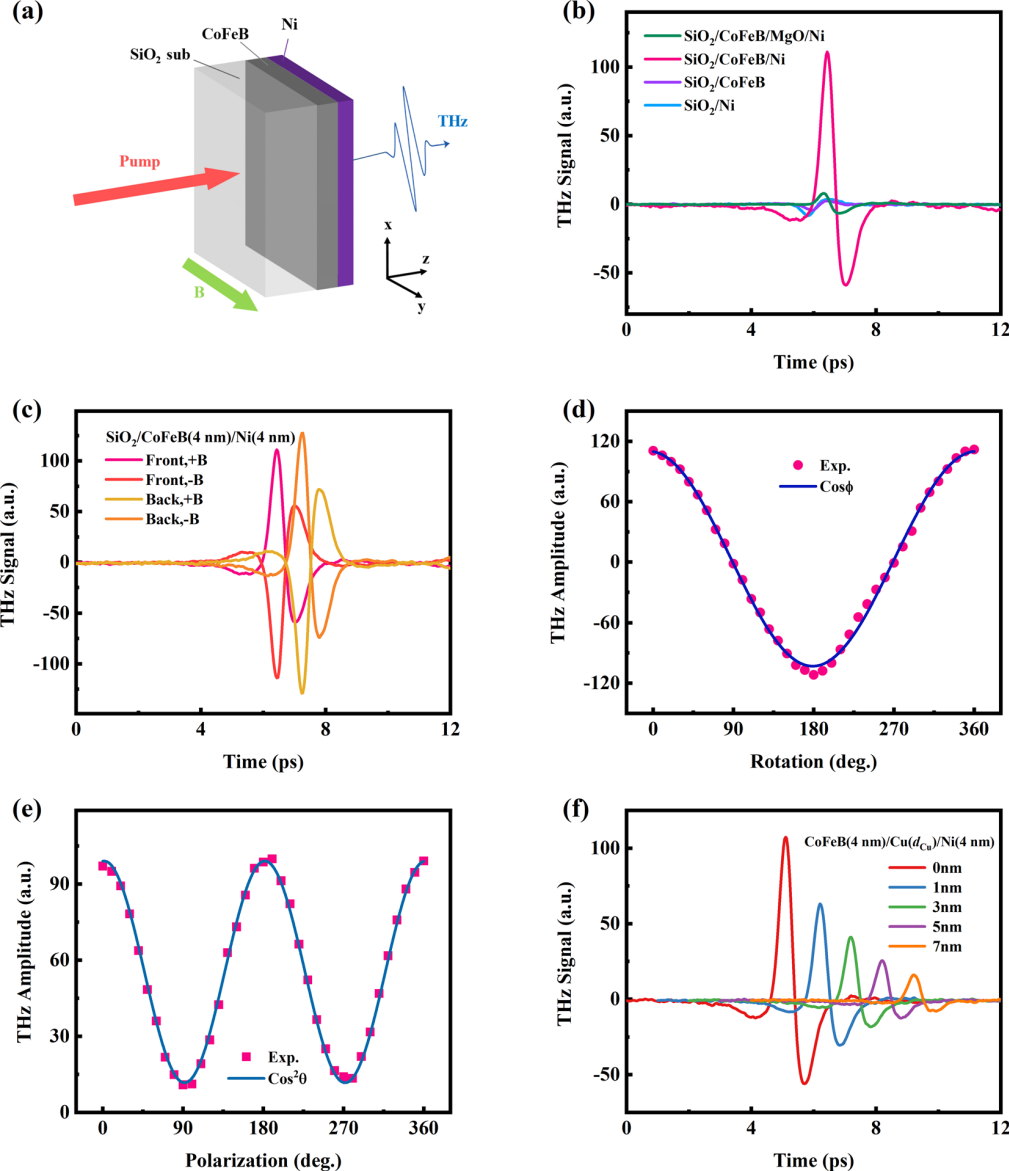
THz emission from CoFeB/Ni bilayer. (a) Schematic of THz emission from CoFeB/Ni bilayer. (b) THz emission from SiO_2_/CoFeB (4 nm)/Ni (4 nm) compared to SiO_2_/CoFeB (4 nm)/MgO(2 nm)/Ni (4 nm), single CoFeB (4 nm) and Ni (4 nm) film. (c) Emitted pulses with reversed magnetic fields and pumping sides. (d) The peak amplitude of THz signal as a function of magnetic field rotation angle *Φ* with respect to positive *y* axis. (e) The peak amplitude of THz signal as a function of polarizer angle *θ*. (f) THz emissions from the CoFeB (4 nm)/Cu (*d*_Cu_)/Ni (4 nm) samples, where *d*_Cu_ = 0, 1, 3, 5 and 7 nm. The data are shifted horizontally for clarity.

As is shown in Figure 3(a), we chose CoFeB as a spin injector and deposited a Ni film on it. Since the SHE of CoFeB is known to be very small [52], the THz SCC from Ni towards CoFeB can be neglected. The pumping and applied external magnetic field are the same as that in Section 1. The typical THz waveforms emitted from the CoFeB (4 nm)/Ni (4 nm) bilayer, single CoFeB (4 nm) and single Ni (4 nm) layer are shown in Figure 3(b). It is noted that compared to the single Ni and CoFeB film, the THz emission intensity from CoFeB/Ni bilayer is significantly stronger, with an opposite polarity. By means of introducing an extra ferromagnetic layer of CoFeB, the THz signal increases an order of magnitude. It is apparent that the enhancement of THz emission results from mechanisms beyond the AHE and ultrafast demagnetization. Thus, it is interesting to investigate the mechanisms in the ferromagnetic CoFeB/Ni bilayer.

As illustrated in Figure 3(c), the THz wave polarity shifts 180° when reversing the magnetic field, indicating the THz wave is strongly dependent on the magnetic field. When flipping the sample, the THz polarity is also reversed due to the reversal of spin current direction. The metal stack order affects the THz emission. It is noted that the amplitude and time delay of THz emissions are different when exciting the samples from the side of substrate and the films, which originates from the different refractive indices of SiO_2_ substrate in 800 nm and THz frequency range. The curve of peak amplitude of THz signal versus the angle *Φ* of rotating magnetic field, as plotted in Figure 3(d), shows a simple cosine curve behavior. Moreover, the THz wave transmits through an analyzing polarizer and its peak amplitude changed with the polarizer rotating angle *θ* is shown in Figure 3(e). This curve follows the cos^2^ *θ* behavior, with the minimum at *θ* = 90° and 270°, showing that the THz electric field is linear and *p* polarized.

Those characteristics are consistent with the behaviors of spintronic THz emission in the FM/NM bilayer. Furthermore, inserting a layer of a MgO can terminate the flow of spin current. As shown in Figure 3(b), the THz emission from SiO_2_/CoFeB (4 nm)/MgO(2 nm)/Ni (4 nm) is strongly attenuated. This proves that the THz wave emitted from CoFeB/Ni bilayer is due to the spin-to-charge conversion between the CoFeB/Ni heterojunction.

For spintronic THz emission, the THz wave is induced by the SCC via either the ISHE in the bulk or the IREE on the surface. For ISHE [15, 16]:

(3) $$j_{\text{c}}\infty \theta_{\text{SHE}}\;j_{\text{s}}\times M,$$

where ***j***_c_ is the charge current, *θ*_SHE_ is the spin Hall angle of NM, ***j***_s_ is spin current and ***M*** is the magnetization of the FM layer. For IREE [36]:

(4) $$j_{\text{c}}\infty \lambda_{\text{IREE}}\;j_{\text{s}}\times \hat{z},$$

where *λ*_IREE_ is the IREE coefficient, $\hat{z}$ is the direction of the potential gradient perpendicular to the interface.

Both two mechanisms can explain why the polarity of THz emission is dependent on the magnetic field direction and the stack order of metal films. Nevertheless, the IREE occurs at the interfaces with broken inversion symmetry and ISHE happens in the bulk of metallic system with spin–orbit coupling (SOC). To distinguish IREE and ISHE, we just need to introduce new interfaces to break the old one. Therefore, we insert a Cu film between the two ferromagnets. Because the spin-diffusion length of Cu film can be a hundred nanometers, spin current can efficiently transmit through the thin Cu film. In this case, the effect of ISHE will not be affected much. Nevertheless, the Cu separator disrupts the CoFeB/Ni interface and impacts significantly the interface-related effect of IREE. The THz emissions of CoFeB (4 nm)/Cu (*d*_Cu_)/Ni (4 nm) are shown in Figure 3(f). When the thickness of Cu layer is increased, the THz signal is attenuated. The decrease of THz signal is ascribed to the loss of spin current in the Cu layer. It is clear that the intensity of THz amplitude is still high in the presence of Cu films. Because the Cu layer is thick enough to destroy the initial interface, the IREE effect on SCC is eliminated. In other words, the ISHE primarily contributes to the THz emission from the CoFeB/Ni bilayer.

**Figure 4:**
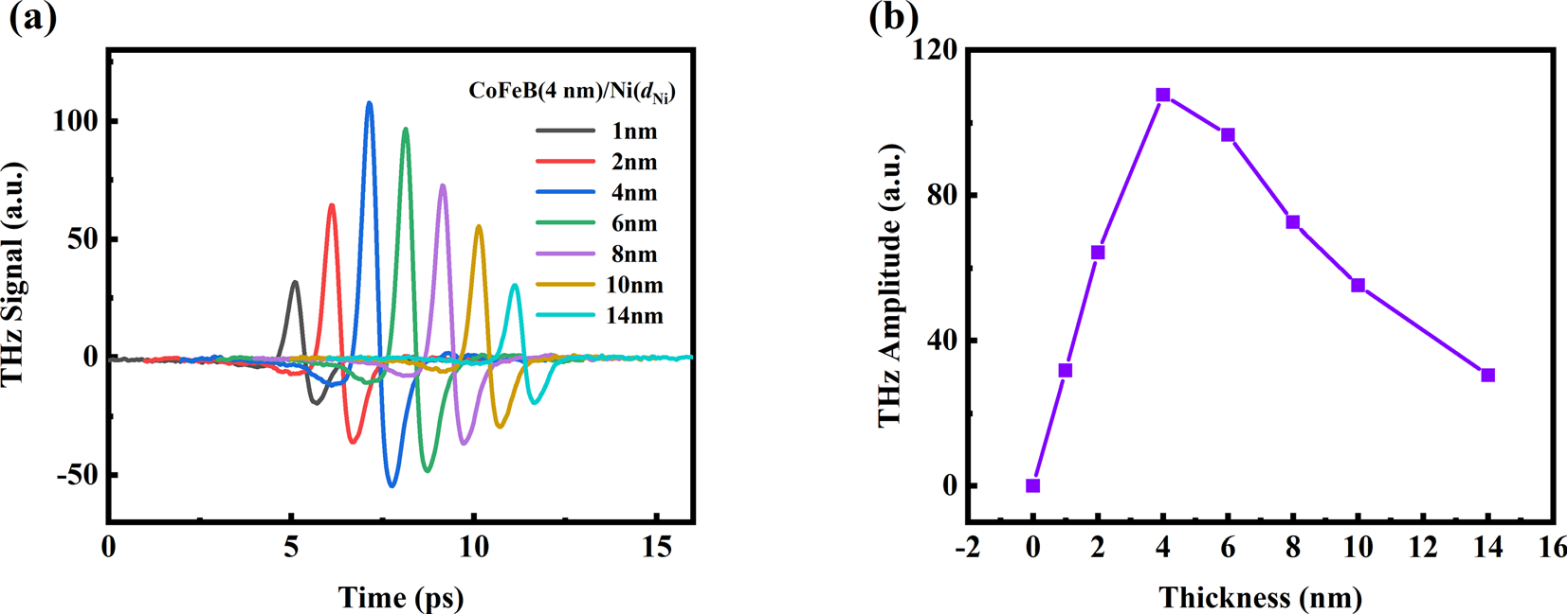
THz signal dependent on Ni thickness in CoFeB/Ni bilayer. (a) THz emission emitted from CoFeB (4 nm)/Ni (*d*_Ni_) bilayer, where *d*_Ni_ = 1, 2, 4, 6, 8, 10 and 14 nm. The data are shifted horizontally for clarity. (b) The THz peak amplitudes dependent on thickness of Ni films for CoFeB (4 nm)/Ni (*d*_Ni_) structures.

Moreover, the dependence of Ni thickness on the THz emission from CoFeB (4 nm)/Ni (*d*_Ni_) bilayer is investigated, as presented in Figure 4. Different from the single Ni metal film, the maximum of THz signal of CoFeB/Ni bilayer occurs at *d*_Ni_ = 4 nm. For the bilayer, the role of Ni is converting the spin currents into charge currents. In this case, the THz signal is dependent on the spin-diffusion of Ni. As Ni thickness increases, spin currents can diffuse further and more charge currents can be generated in the bulk, increasing terahertz emission. However, on the other hand, the sample impedance decreases with the increasing Ni thickness, leading to more absorption of THz wave by the metal films, and thus reducing THz emission.

In conclusion, the ISHE is the mechanism for the strong THz SCC in CoFeB/Ni structure. Beside the ISHE, as shown in Figure 3(b), the single layer of CoFeB and Ni ferromagnet film could also emit THz radiation. As illustrated in Part 1, the THz emission from single ferromagnet film is the co-effect of AHE and ultrafast demagnetization. However, the strength of THz signal from the single CoFeB and Ni layer is an order of magnitude smaller than that of CoFeB/Ni bilayer. Therefore, in CoFeB/Ni bilayer, the THz emission consists of those parts: (1) the contributions of co-effect of AHE and ultrafast demagnetization in CoFeB and Ni films, respectively. (2) The ISHE in ferromagnetic Ni film. And the ISHE is dominant in above mechanisms.

Because the Ni is ferromagnetic metal, it can also be acted as a spin injector in FM/NM bilayer, which is further investigated in the following section.

### 2.3 Terahertz emission from Ni/NM structure

The spintronic THz emission has been widely studied on FM/NM structure via SCC. The major spin-to-charge effects are realized by ISHE and IREE. As mentioned in the above section, the former occurs in the bulk while the latter is occurring on the surface with broken inversion symmetry. Recently, interfacial skew scattering in FM/NM bilayer has also been shown to realize this SCC. In this part, we study the THz wave emitted from the Ni/Pt structure. In this case, the Ni layer acts as a spin injector, and the SCC in Pt film ascribes to the ISHE.

**Figure 5:**
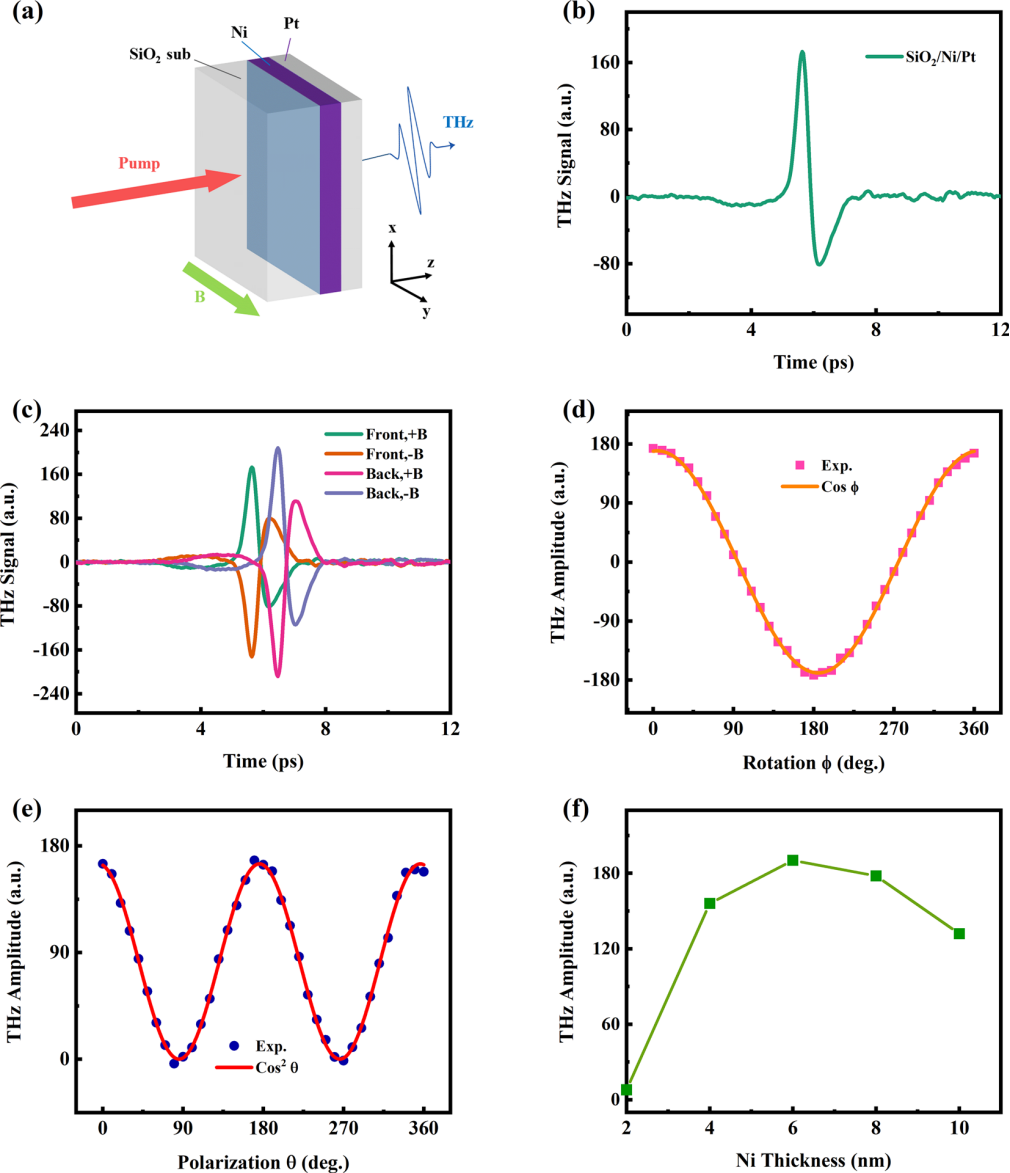
THz emission from Ni/Pt bilayer. (a) Schematic of THz emission from Ni/Pt bilayer. THz emission from Ni (4 nm)/Pt (4 nm) bilayer: (b) Typical emitted pulse. (c) Emitted pulses measured with pumping from the side of substrate (front) and films (back) and reversed magnetic fields. (d) The peak amplitude of THz signal as a function of magnetic field rotation angle *Φ* with respect to positive *y* axis. (e) The peak amplitude of THz signal as a function of polarizer angle *θ*. (f) The peak amplitude of THz signal dependent on the Ni thickness.

Figure 5(a) shows the schematic of THz wave generated from the Ni/Pt bilayer. Under the SiO_2_/Ni structure, we grew a 4 nm Pt film on the top of Ni layer. The pump laser is incident from the SiO_2_ substrate, and the applied magnetic field is in positive direction. The typical THz waveform emitted from Ni (4 nm)/Pt (4 nm) bilayer is shown in Figure 5(b). The THz wave emitted from the Ni/Pt structure is comparable to that of CoFeB/Ni bilayer, and the polarity of THz wave is also the same. Furthermore, as illustrated in Figure 5(c), when reversing the magnetic field or flipping the sample, the THz polarity is also reversed, indicating the THz wave is strongly dependent on the magnetic field and metal stack order. The curve of peak amplitude of THz signal versus the angle *Φ* of rotating magnetic field, as plotted in Figure 5(d), shows a simple cosine curve behavior. Moreover, the THz wave transmits through an analyzing polarizer and its peak amplitude as a function of the polarizer rotating angle *θ* is shown in Figure 5(e). This curve follows the cos^2^ *θ* behavior, with the minimum at *θ* = 90° and 270°, showing that the THz wave field is linear and *p* polarized.

Those behaviors are common in the FM/NM bilayer and when the NM layer is Pt film, the SCC is performed by the ISHE. For ISHE, expression (3) explains why the polarity of THz emission is dependent on the magnetic field direction and the stack order of metal films. The process of THz emission from the Ni/Pt structure can be described as follows. Firstly, the femtosecond pulse injects to the Ni layer, causing demagnetization and exciting spin-polarized currents due to the different mobility of the majority and minority spins. Then the spin-polarized currents reach to the Pt film and transform to the ultrafast charge currents by the ISHE, leading to THz emission.

Furthermore, the influence of thickness of Ni film on the THz emission is also studied, with results shown in Figure 5(f). Similar to the single Ni structure, the THz amplitude reaches the maximum at 6 nm of Ni thickness and decreases when Ni thickness exceeding 6 nm. This result is mainly related to the saturation magnetization of Ni films, laser-induced spin diffusion and THz optical absorption in Ni films.

However, the AHE and ultrafast demagnetization is also existed in the Ni/Pt bilayer. So, there are three mechanisms contribute to the THz emission in the Ni/Pt structure. Similar to the CoFeB/Ni bilayer, the ISHE is the main mechanism for the THz radiation.

### 2.4 Terahertz emission from FM/Ni/NM structure

**Figure 6:**
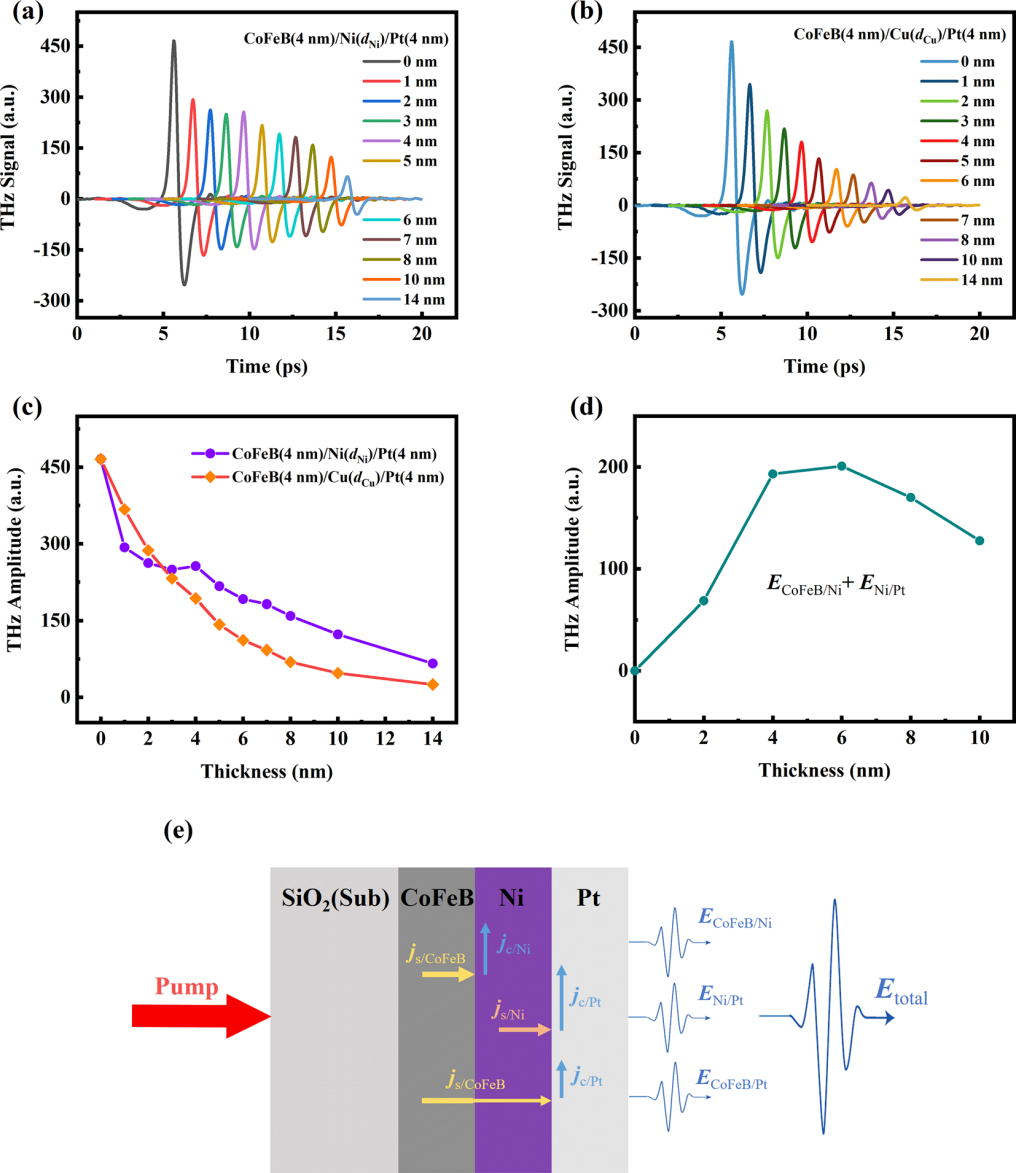
THz emission from CoFeB/Ni/Pt trilayer. (a) THz emission emitted from CoFeB (4 nm)/Ni (*d*_Ni_)/Pt (4 nm) trilayer, where *d*_Ni_ = 0, 1, 2, 3, 4, 5, 6, 7, 8, 10 and 14 nm. The data are shifted horizontally for clarity. (b) THz emission emitted from CoFeB (4 nm)/Cu (*d*_Cu_)/Pt (4 nm) trilayer, where *d*_Cu_ = 0, 1, 2, 3, 4, 5, 6, 7, 8, 10 and 14 nm. The data are shifted horizontally for clarity. (c) The THz peak amplitudes dependent on thickness of Ni and Cu films for trilayer structures. (d) The values of ***E***_CoFeB/Ni_ + ***E***_Ni/Pt_ calculated from Figures 3(b) and 4(f). (e) Schematic of THz emission from CoFeB/Ni/Pt trilayer.

From Part 2 and Part 3, we find the polarity of THz wave emitted from the CoFeB/Ni bilayer and Ni/Pt bilayer is the same. Thus, it is natural to think of combining two structures as a trilayer structure. So, we further study the THz emission from CoFeB/Ni/Pt trilayer, with results shown in Figure 6(a). We fixed the thickness of CoFeB and Pt layer to be 4 nm and changed the Ni thickness. Moreover, we used the Cu films to replace the Ni films for comparison, as shown in Figure 6(b). The dependence of THz peak amplitude on the thickness of Ni and Cu films is presented in Figure 6(c). It is found the THz signals emitted from the CoFeB (4 nm)/Cu (*d*_Cu_)/Pt (4 nm) trilayer decrease smoothly with the increasing thickness of Cu layer. However, for the CoFeB (4 nm)/Ni (*d*_Ni_)/Pt (4 nm) trilayer, the THz signal does not decrease smoothly, but even shows an increasing tendency at *d*_Ni_ = 4 nm. This can be explained as follows.

Because the THz signals from co-effect of AHE and ultrafast demagnetization are quite small compared to the ISHE. To simplify the process in trilayer, we neglect the THz emission from each single FM layer. Therefore, the THz signal ***E***_total_ from the CoFeB/Ni/Pt trilayer can be divided into three parts: (1) THz signal ***E***_CoFeB/Ni_ emitted from CoFeB/Ni structure via ISHE. (2) THz signal ***E***_Ni/Pt_ emitted from Ni/Pt structure. (3) THz signal ***E***_CoFeB/Pt_, this process is the spin currents excited in the CoFeB layer and pass through the Ni film to the Pt layer, and then convert into the charge currents, radiating THz wave.

The spin diffusion length of Cu is quite long even over hundreds nanometers [42], thus, the spin current excited from CoFeB layer can easily pass through the Cu layer and transform to charge current. The THz signals of CoFeB/Cu/Pt trilayer is from ***E***_CoFeB/Pt_. This is why the THz signals of CoFeB/Cu/Pt trilayer drops smoothly. However, the spin diffusion length of Ni is reported about 3.3 nm in Ref [53]. As a result, when the Ni thickness over this length, the spin current generated from CoFeB layer is depleted in Ni layer. Therefore, when the Ni thickness over 3 nm, the contribution of ***E***_CoFeB/Pt_ is non-existent or very small. The THz emission of CoFeB/Ni/Pt trilayer is composed of ***E***_CoFeB/Ni_ and ***E***_Ni/Pt_. And their contribution is comparable according to Figures 4(b) and 5(f). We calculated roughly the values of ***E***_CoFeB/Ni_ + ***E***_Ni/Pt_ at each thickness from the figures in Figures 4(b) and 5(f), and the results is shown in Figure 6(d). When the Ni thickness over 6 nm, the values of ***E***_CoFeB/Ni_ + ***E***_Ni/Pt_ decreases as the Ni thickness increases, so dose ***E***_total_. When Ni thickness is less than 3 nm, the value of ***E***_CoFeB/Ni_ + ***E***_Ni/Pt_ is much smaller than the ***E***_total_. This means that most of ***E***_total_ comes from ***E***_CoFeB/Pt_.

In general, as is shown in Figure 6(e), the THz signal *E*_total_ from the CoFeB/Ni/Pt trilayer contains three parts: ***E***_CoFeB/Ni_, ***E***_Ni/Pt_ and ***E***_CoFeB/Pt_. When the Ni thickness less than 3 nm, the contribution of ***E***_CoFeB/Pt_ is dominant. The ***E***_CoFeB/Ni_ and ***E***_Ni/Pt_ are in dominant when the Ni thickness over 3 nm, and their contribution is comparable. So in theory, if we want to enhance the signal through the FM1/FM2/NM trilayers, several conditions need to be met. Firstly, the SHA of FM2 should be large. Then, the spin diffusion length of FM2 should be long. And the THz emission should be efficient when FM2 in thin thickness. We believe this FM2 materials should be found in the further woks.

## 3 Conclusions

In summary, we have studied the THz spin-to-charge conversion of ferromagnetic Ni thin film in various types of structures. It is found that the Ni film has different functions in the SCC process. In the single Ni layer structure, the THz emission is radiated by the AHE and ultrafast demagnetization of ferromagnetic Ni thin film. In the FM/Ni (CoFeB/Ni) structure, the Ni film acts as an SCC implementer and the THz emission is mainly generated by the ISHE of the Ni film. In the Ni/NM (Ni/Pt) structure, the Ni film acts as a spin injector and provides spin currents, which can be converted to charge current via ISHE in heavy metal film, inducing THz emission. Moreover, we have also studied the THz emission from the FM/Ni/NM trilayer (CoFeB/Ni/Pt) and explored the possibility of enhancing the THz wave. In theory, for an FM1/FM2/NM structure, if the SHA of FM2 is large enough and the spin diffusion length of FM2 is long enough, it is greatly possible to achieve an enhanced THz emission signal. We demonstrate that the ferromagnetic metals can have diverse functions in the THz SCC process, which shows great potential in the spintronic THz emitters.

## 4 Experimental section

### 4.1 Sample preparation

All films were grown on a 2-polished and 500 μm-thick SiO_2_ substrate by magnetron sputtering with a pressure of sputtering chamber below 2 × 10^−6^ mTorr. The Co_40_Fe_40_B_20_ (CoFeB) film is directly sputtered from the Co_40_Fe_40_B_20_ target acted as a spin current injector.

### 4.2 Terahertz emission spectroscopy

A Ti: Sapphire laser with a pulse width of 35 fs, a center wavelength of 800 nm and a repetition rate of 1 kHz is used in our experiment. The laser beam was split into two parts: detection part and excitation part. The excited beam was focused on the sample with spot size of about 3 mm in diameter and the laser power was 100 mW. The THz emission was collected by a pair of parabolic mirrors and then focused onto a 500 μm-thick ZnTe (110) crystal. After the detection beam passed through the ZnTe crystal, it experienced the THz electric field-induced polarization rotation due to the electro-optical effect, which could be analyzed by using a quarter-wave plate, a Wollaston prism, and a balanced detector. Before measuring THz emission signals, the sample were magnetized by a pair of magnets with magnetic field (70 mT) along the *y*-axis.
